# Resting state functional brain networks associated with emotion processing in frontotemporal lobar degeneration

**DOI:** 10.1038/s41380-022-01612-9

**Published:** 2022-05-20

**Authors:** Elisa Canu, Davide Calderaro, Veronica Castelnovo, Silvia Basaia, Maria Antonietta Magno, Nilo Riva, Giuseppe Magnani, Francesca Caso, Paola Caroppo, Sara Prioni, Cristina Villa, Debora Pain, Gabriele Mora, Lucio Tremolizzo, Ildebrando Appollonio, Barbara Poletti, Vincenzo Silani, Massimo Filippi, Federica Agosta

**Affiliations:** 1 Neuroimaging Research Unit, Division of Neuroscience, IRCCS San Raffaele Scientific Institute, Milan, Italy; 2 Vita-Salute San Raffaele University, Milan, Italy; 3 Neurorehabilitation Unit, IRCCS San Raffaele Scientific Institute, Milan, Italy; 4 Experimental Neuropathology Unit, Division of Neuroscience, IRCCS San Raffaele Scientific Institute, Milan, Italy; 5 Neurology Unit, IRCCS San Raffaele Scientific Institute, Milan, Italy; 6 Fondazione IRCCS Istituto Neurologico Carlo Besta, Unit of Neurology 5—Neuropathology, Milan, Italy; 7 Istituti Clinici Scientifici Maugeri, IRCCS, Neurorehabilitation Department of Milano Institute, Milan, Italy; 8 Neurology Unit, “San Gerardo” Hospital and University of Milano-Bicocca, Monza, Italy; 9 Department of Neurology and Laboratory of Neuroscience, IRCCS Istituto Auxologico Italiano, Milan, Italy; 10 “Dino Ferrari” Center, Department of Pathophysiology and Transplantation, Università degli Studi di Milano, Milan, Italy; 11 Neurophysiology Service, IRCCS San Raffaele Scientific Institute, Milan, Italy

**Keywords:** Neuroscience, Predictive markers

## Abstract

This study investigated the relationship between emotion processing and resting-state functional connectivity (rs-FC) of the brain networks in frontotemporal lobar degeneration (FTLD). Eighty FTLD patients (including cases with behavioral variant of frontotemporal dementia, primary progressive aphasia, progressive supranuclear palsy syndrome, motor neuron disease) and 65 healthy controls underwent rs-functional MRI. Emotion processing was tested using the Comprehensive Affect Testing System (CATS). In patients and controls, correlations were investigated between each emotion construct and rs-FC changes within critical networks. Mean rs-FC of the clusters significantly associated with CATS scoring were compared among FTLD groups. FTLD patients had pathological CATS scores compared with controls. In controls, increased rs-FC of the cerebellar and visuo-associative networks correlated with better scores in emotion-matching and discrimination tasks, respectively; while decreased rs-FC of the visuo-spatial network was related with better performance in the affect-matching and naming. In FTLD, the associations between rs-FC and CATS scores involved more brain regions, such as orbitofrontal and middle frontal gyri within anterior networks (i.e., salience and default-mode), parietal and somatosensory regions within visuo-spatial and sensorimotor networks, caudate and thalamus within basal-ganglia network. Rs-FC changes associated with CATS were similar among all FTLD groups. In FTLD compared to controls, the pattern of rs-FC associated with emotional processing involves a larger number of brain regions, likely due to functional specificity loss and compensatory attempts. These associations were similar across all FTLD groups, suggesting a common physiopathological mechanism of emotion processing breakdown, regardless the clinical presentation and pattern of atrophy.

## Introduction

Among the social cognitive functions, the perception of social stimuli is a highly developed skill, gathering crucial information for interpersonal communication. The capacity to associate specific patterns of facial musculature contractions to discrete emotions is an universal aspect of social communication, equally recognized across different cultures [1]. To evaluate emotion recognition, the most commonly used stimuli are the Ekman’s pictures of facial affect, a collection of photos to investigate an individual’s ability to discriminate and label the six basic emotions (disgust, surprise, happiness, anger, fear and sadness) [2]. Defective emotion recognition can lead to altered social interactions, especially in disorders affecting the frontal and the temporal lobes, such as those belonging to the frontotemporal lobar degeneration (FTLD) spectrum. Specifically, patients with the behavioral variant of frontotemporal dementia (bvFTD) [3], agrammatic/non-fluent (nfvPPA) and semantic (svPPA) variants of primary progressive aphasia (PPA) [4, 5], progressive supranuclear palsy syndrome (PSPs) [6] and amyotrophic lateral sclerosis (ALS) [7, 8], all show reduced emotional reaction and/or recognition mainly for negative stimuli [3]. Subtle affect processing failures are already present in presymptomatic *C9orf72* mutation carriers at risk for bvFTD, as compared with both controls and carriers of other mutations [9, 10].

A set of brain regions, involving limbic and primary sensory systems, are crucial for a rapid and automatic evaluation of the perceived emotion and functional MRI (fMRI) studies showed that they are also engaged during non-conscious subliminal perception of affective stimuli [11]. Emotion identification deficits in FTLD patients have been linked to decreased gray matter (GM) volume of amygdala, insula, inferior frontal, medial prefrontal and orbitofrontal cortices, with a prevalent involvement of the right side, as well as with diffusivity abnormalities of the right inferior longitudinal and inferior fronto-occipital fasciculi, and fornix [12, 13]. Functionally, when viewing videos of emotional facial expressions during task-based fMRI paradigms, FTLD patients have been shown to activate less the fusiform gyrus [14].

Although the interest of the scientific community in investigating social cognition has significantly increased over the past few years, the neural functional correlates of social cognitive deficits in patients affected by FTLD are still not fully established. In this study, we aimed at investigating the relationship between emotion processing and the functional connectivity of the resting state (rs-FC) brain networks in healthy controls and a large cohort of FTLD patients. To this purpose, we used the Comprehensive Affect Testing System (CATS) battery, which investigates different aspects of the emotion processing using the Ekman’s pictures of facial affect. Furthermore, we wished to understand whether the link between emotion processing and brain functional connectivity is differently modulated by the disease phenotype or is shared by all FTLD clinical variants, regardless the clinical presentation, pattern of atrophy and genetic status.

We expected that all FTLD patients would present lower CATS performances when compared to healthy controls, and that, among FTLD groups, bvFTD and motor neuron disorder (MND) cases would obtain the worst and the best CATS scores, respectively. In agreement with these clinical prediction, we supported the hypothesis of a *‘trans-disease’* model. According to this hypothesis, in different forms of FTLD in which emotion recognition is impaired, we expected to obtain rs-FC findings that go beyond the single clinical entity and rather reflect common brain processing failure in these conditions.

## Methods

A total of 144 patients with a suspected diagnosis of FTLD-related disorders were prospectively enrolled at five referral clinics in Lombardy, Italy and referred to San Raffaele Hospital in Milan between May 2017 and January 2020 to perform MRI on a 3 T scanner, as part of their diagnostic work-up. Among them, we selected patients who: received a clinical diagnosis of FTLD clinical variant (i.e., probable bvFTD [6], probable nfvPPA and svPPA [15], PSPs [16] or MND including ALS [17], primary lateral sclerosis [PLS] [18], progressive muscular atrophy [PMA] [19]); gave consent to be screened for known pathogenic mutations; performed clinical assessment, neuropsychological battery including an evaluation of emotion processing (see details below), and brain 3 T T1-weighted and rs-fMRI. The final cohort included 66 sporadic FTLD cases (18 bvFTD, 12 PPA [5 nfvPPA and 7 svPPA], 10 PSPs, 26 MND [20 ALS, 3 PLS and 3 PMA]) and 14 FTLD mutation carriers. Genetic cases included 8 bvFTD (g-bvFTD: 3 *C9orf72*, 3 *GRN*, 1 *MAPT*, 1 *TREM2*) and 6 MND (g-MND = 5 ALS: 3 *C9orf72*, 1 *TARDBP*, 1 *SOD1*; 1 PMA: 1 *TARDBP*). Eighteen patients (13 bvFTD, 2 g-bvFTD, 4 PPA, 4 PSPs) also underwent lumbar puncture to exclude cerebrospinal fluid biomarker profile suggestive of Alzheimer’s disease pathology, as part of their diagnostic work-up [20]. Table 1 summarizes demographic and clinical features of included subjects.

**Table 1:** Demographic, clinical and neuropsychological features of patients and healthy controls.

	HC	FTLD	*p*-value	bvFTD	g-bvFTD	PPA	PSP	MND	g-MND
***N***	65	80	–	18	8	12	10	26	6
**Age [years]**	61.83 ± 8.92 (40.2–83.4)	61.93 ± 11.16 (23.8–84.1)	0.95	64.64 ± 8.43 (45.9–78.6)	60.02 ± 6.51 (48.6–66.7)	61.79 ± 10.00 (47.8–78.0)	69.02 ± 7.95 (59.5–84.1)	58.15 ± 14.51 (23.8–78.9)	61.26 ± 8.37 (49.1–70.7)
**Sex [women/men]**	46/19	33/47	<0.001	4/14	2/6	6/6	7/3	11/15	3/3
**Education [years]**	11.83 ± 3.86 (5.0–21.0)	11.44 ± 4.24 (5.0–28.0)	0.56	10.67 ± 2.45 (8.0–13.0)	10.38 ± 3.11 (5.0–13.0)	12.42 ± 5.55 (5.0–21.0)	10.20 ± 5.10 (5.0–23.0)	12.69 ± 4.70 (5.0–28.0)	9.83 ± 2.14 (8.0 + 13.0)
**Disease duration [months]**	–	44.84 ± 43.53 (0.5–211.0)	–	48.83 ± 26.76 (13.9–107.6)	59.06 ± 43.41 (13.0–140.2)	36.64 ± 37.03 (14.0–132.8)	31.68 ± 11.89 (14.4–53.4)	50.42 ± 64.22 (0.5–211.0)	24.26 ± 27.75 (4.8–78.0)
**CDR-FTD**	–	6.76 ± 5.60 (0.0–22.0)	–	8.21 ± 5.31 (1.0–20.0)	12.58 ± 7.18 (3.0–22.0)	3.70 ± 2.19^**a**^ (1.0–7.5)	4.43 ± 3.72^**a**^ (1.0–11.5)	–	–
**Global cognition**									
MMSE	29.36 ± 0.84 (27.0–30.0)	25.82 ± 4.71 (6.0–30.0)	<0.001	23.29 ± 5.99^**b**^ (6.0–30.0)	20.88 ± 6.62^**b**^ (10.0–27.0)	25.27 ± 3.72^**b**^ (17.0–30.0)	26.10 ± 2.28^**a**^ (22. 0–29. 0)	28.42 ± 1.98^a,^^**c**^ (24.0–30.0)	28.83 ± 1.17^a,^^**c**^ (27.0–30.0)
FAB	–	11.80 ± 3.84 (0.0–17.0)	–	11.73 ± 4.68 (0.0–17.0)	8.17 ± 4.07 (2.0–13.0)	13.80 ± 2.10 (11.0–17.0)	10.20 ± 2.39 (6.0–13.0)	14.57 ± 2.23 (11.0–17.0)	11.50 ± 3.54 (9.0–14.0)
**Verbal memory**									
Digit span, forward	5.88 ± 0.93 (4.0–8.0)	5.10 ± 1.35 (2.0–8.0)	<0.001	5.00 ± 1.28 (3.0–7.0)	3.63 ± 1.19^**b**^ (2.0–6.0)	5.27 ± 0.79 (4.0–6.0)	4.80 ± 0.92 (3.0–6.0)	5.80 ± 1.47^**a**^ (3.0–8.0)	4.67 ± 1.03 (3.0–6.0)
RAVLT, delayed recall	10.70 ± 2.33 (4.0–15.0)	6.23 ± 4.0 (0.0–15.0)	<0.001	3.38 ± 2.83^**b**^ (0.0–8.0)	3.29 ± 2.75^**b**^ (0.0–7.0)	4.44 ± 4.07^**b**^ (0.0–10.0)	4.60 ± 2.01^**b**^ (2.0–9.0)	9.62 ± 3.09^a,^^**c,d,e**^ (3.0–15.0)	8.0 ± 2.53 (5.0–12.0)
**Spatial memory**									
Spatial span, forward	5.26 ± 1.09 (3.0–7.0)	3.98 ± 1.38 (0.0–7.0)	<0.001	4.33 ± 1.33^**b**^ (2.0–7.0)	2.86 ± 1.86^**b**^ (0.0–5.0)	4.33 ± 1.44 (2.0–7.0)	3.80 ± 0.63 (3.0–5.0)	–	–
Benson’s figure, recall	11.30 ± 3.21 (5.0–17.0)	6.11 ± 3.85 (0.0–16.0)	<0.001	5.31 ± 3.44^**b**^ (0.0–12.0)	5.43 ± 4.04^**b**^ (0.0–10.0)	8.08 ± 4.74 (0.0–16.0)	5.70 ± 2.95^**b**^ (1.0–10.0)	–	–
**Executive functions**									
Digit span, backward	4.75 ± 1.08 (3.0–8.0)	3.63 ± 1.82 (0.0–9.0)	<0.001	3.56 ± 1.90 (0.0–8.0)	2.25 ± 1.49^**b**^ (0.0–4.0)	2.80 ± 1.40^**b**^ (0.0–5.0)	2.56 ± 1.59^**b**^ (0.0–5.0)	4.67 ± 1.63^**a**^ (1.0–9.0)	4.50 ± 1.23 (3.0–6.0)
MCST, perseverations	3.28 ± 3.17 (0.0–13.0)	9.79 ± 10.92 (0.0–47.0)	<0.001	14.87 ± 14.07^**b**^ (0.0–47.0)	14.80 ± 5.26 (7.0–20.0)	6.00 ± 8.85 (0.0–25.0)	13.63 ± 14.38 (1.0–47.0)	5.84 ± 8.23 (0.0–29.0)	8.67 ± 6.35 (1.0–16.0)
**Language**									
Token test	34.05 ± 1.75 (30.0–36.0)	27.57 ± 6.51 (5.0–36.0)	<0.001	27.22 ± 7.32^**b**^ (5.0–35.0)	22.25 ± 6.84^**b**^ (12.0–33.0)	27.00 ± 6.87^**b**^ (15.5–36.0)	27.95 ± 4.95^**b**^ (18.5–36.0)	–	–
Phonemic Fluency	36.66 ± 8.33 (18.0–59.0)	22.81 ± 14.61 (0.0–59.0)	<0.001	17.59 ± 10.70^**b**^ (0.0–39.0)	8.13 ± 7.61^**b**^ (0.0–24.0)	17.09 ± 9.27^**b**^ (3.0–32.0)	14.40 ± 7.62^**b**^ (5.0–27.0)	34.58 ± 13.62^a,^^**c,d,e**^ (11.0–59.0)	30.67 ± 13.78^**a,e**^ (20.0–55.0)
**Social cognition**									
CATS, Total score	56.34 ± 5.16 (45.0–69.0)	46.66 ± 3.85 (25.0–67.0)	<0.001	43.61 ± 7.16^**b**^ (32.0–55.0)	38.00 ± 7.73^**b**^ (25.0–50.0)	45.92 ± 8.94^**b**^ (36.0–65.0)	43.60 ± 6.50^**b**^ (35.0–53.0)	51.31 ± 6.44^a,**c**^ (40.0–67.0)	49.50 ± 6.06 (43.0–58.0)
CATS, Affect discrimination	11.28 ± 0.94 (7.0–12.0)	10.56 ± 1.61 (6.0–12.0)	0.03	10.00 ± 2.11^**b**^ (6.0–12.0)	8.88 ± 1.73^**b**^ (6.0–11.0)	10.58 ± 1.00 (9.0–12.0)	10.40 ± 1.90 (7.0–12.0)	11.27 ± 0.78^**a**^ (10.0–12.0)	11.67 ± 0.52^**a**^ (11.0–12.0)
CATS, Affect naming	4.62 ± 1.00 (2.0–6.0)	3.44 ± 1.59 (0.0–6.0)	<0.001	2.89 ± 1.28^**b**^ (0.0–5.0)	2.13 ± 1.55^**b**^ (0.0–5.0)	3.25 ± 1.77 (0.0–6.0)	3.20 ± 1.69 (0.0–5.0)	4.19 ± 1.39^**a**^ (1.0–6.0)	4.33 ± 1.21 (3.0–6.0)
CATS, Affect matching	9.12 ± 1.88 (5.0–12.0)	6.96 ± 1.95 (2.0–12.0)	<0.001	6.67 ± 1.65^**b**^ (4.0–10.0)	5.25 ± 1.58^**b**^ (2.0–7.0)	6.83 ± 2.73^**b**^ (2.0–11.0)	6.60 ± 1.57 (5.0–9.0)	7.85 ± 1.76 (5.0–12.0)	7.00 ± 1.41 (5.0–9.0)
SET, Global score	–	12.32 ± 3.97 (3.0–18.0)	–	10.38 ± 3.98 (3.0–16.0)	10.50 ± 3.42 (7.0–15.0)	11.82 ± 3.66 (5.0–16.0)	11.67 ± 4.98 (3.0–17.0)	14.15 ± 3.32 (5.0–18.0)	12.67 ± 3.72 (7.0–16.0)
**Visuospatial abilities**									
Benson’s figure, copy	15.73 ± 0.73 (14.0–17.0)	12.77 ± 3.44 (0.0–16.0)	<0.001	13.12 ± 4.05^**b**^ (0.0–16.0)	11.29 ± 4.35^**b**^ (5.0–16.0)	13.92 ± 2.39 (9.0–16.0)	11.30 ± 2.26^**b**^ (9.0–15.0)	–	–
Copy of drawings	67.22 ± 3.64 (56.0–70.0)	62.38 ± 6.72 (46.0–70.0)	<0.001	62.88 ± 5.55 (54.0–70.0)	62.86 ± 7.84 (50.0–70.0)	64.42 ± 7.45 (51.0–70.0)	57.50 ± 5.99^**b**^ (46.0–68.0)	–	–
**Behavior**									
FBI, Total	–	13.63 ± 13.42 (0.0–51.0)	–	27.36 ± 11.39 (13.0–51.0)	27.60 ± 14.31 (11.0–49.0)	12.83 ± 11.72^**c**^ (3.0–37.0)	13.50 ± 7.19 (3.0–27.0)	3.42 ± 5.36^a,^^c^ (0.0–19.0)	2.20 ± 1.64^a,^^**c**^ (1.0–5.0)
NPI	–	16.71 ± 21.20 (0.0–102.0)	–	28.82 ± 21.88 (6.0–76.0)	30.00 ± 34.60 (4.0–12.0)	16.58 ± 20.81 (0.0–71.0)	11.40 ± 8.53 (2.0–23.0)	4.94 ± 6.80^c^ (0.0–28.0)	2.60 ± 2.07 (0.0–5.0)

Values are numbers or means ± standard deviations (range). Disease duration was defined as months from onset to date of MRI scan. The number of patients performing each test is reported in the Table. *P* values refer to ANOVA models, followed by post-hoc pairwise comparisons (Bonferroni-corrected for multiple comparisons), or Chi-square test. Differences in neuropsychological scores were assessed using ANCOVA models, accounting for age, sex and education, and followed by post-hoc pairwise comparisons (Bonferroni-corrected for multiple comparisons).

*bvFTD* behavioral variant of frontotemporal dementia, *CATS* Comprehensive Affect Testing System, *CDR-FTD* clinical dementia rating scale for frontotemporal dementia, *FAB* frontal assessment battery, *FBI* frontal behavioral inventory, *HC* healthy controls, *g-bvFTD* behavioral variant of frontotemporal dementia with genetic mutation, *MCST* modified card sorting tests, *MMSE* mini-mental state examination, *MND* motor neuron disorder, *g-MND* motor neuron disorder with genetic mutation, *N* Number, *NPI* neuropsychiatric inventory, *PSP* progressive supranuclear palsy, *RAVLT* Rey auditory verbal learning test, *SET* story-based empathy task.

Significance was considered for *p* values lower than 0.05

^a^vs g-bvFTD.

^b^vs healthy controls.

^c^vs bvFTD.

^d^vs PPA.

^e^vs PSP.

Sixty-five healthy controls comparable with patients for age and education were recruited by word of mouth among subjects unrelated to the patient population. An independent group of 33 young healthy controls (age: 24.9 ± 2.8 years; 14 [42%] women; education: 15.4 ± 3.1 years) were also recruited among students at Vita-Salute San Raffaele University in Milan in order to generate independent components (ICs) of interest for the rs-fMRI analysis (see details below). All controls were recruited based on the following criteria: no family history of neurodegenerative diseases, and normal neurological and cognitive assessment (Table 1).

Exclusion criteria for all subjects were: medical illnesses or substance abuse that could interfere with cognitive functioning; any (other) major systemic, psychiatric, or neurological illnesses; and other causes of focal or diffuse brain damage, including lacunae and extensive cerebrovascular disorders at routine MRI.

### Standard protocol approvals, registrations, and patient consents

The local ethical standards committee on human experimentation of IRCCS Ospedale San Raffaele approved the study protocol and all participants provided written informed consent.

### Clinical evaluation

Clinical evaluations were performed by experienced neurologists blinded to MRI results. For all patients, excluding MND cases, disease severity was assessed using the CDR-FTD [21] and independence with basic and instrumental activities of daily life [22, 23]. For MND patients, disease severity was assessed using the ALSFRS-r [24] and the rate of disease progression was defined according to the formula: [48–ALSFRS-r score]/time from symptom onset. This formula has been adapted from Ellis et al. [25], and it has been developed since it expresses the ALSFRS-r as a function of the disease duration, thus providing indication of disease rapidity [26]. In different studies, this formula has been demonstrated to be a reliable prognostic biomarker of MND evolution [26–29].

### Cognitive and behavioral assessment

Neuropsychological assessments were performed by experienced neuropsychologists, unaware of MRI results. In all patients, emotion processing was evaluated using the CATS [30], which investigates different aspects of the emotion processing using the Ekman’s pictures of facial affect expressing the six basic emotions (disgust, surprise, happiness, anger, fear and sadness). From this battery, we selected and administered the following subtests: affect discrimination (12 trials in which the patient is required to state whether two presented faces express the same or different emotions), affect naming (6 trials in which the patient is required to select, among 7 possible choices, the emotional label that best describes the emotion expressed by the face target), and affect matching (12 trials in which the patient is required to select, among 5 possible facial affect pictures, which is the one expressing the same emotion of the face target) (Supplementary Fig. 1). Based on the number of correct answers, we obtained a total score and specific scores for each CATS subdomain.

The following cognitive functions were also investigated, as previously described [31]: global cognitive functioning with the MMSE [32] and the frontal assessment battery (FAB) [33]; long and short term verbal memory with the Rey Auditory Verbal Learning Test [34] and the digit span forward [35], respectively; long and short term spatial memory with the recall and recognition of the Benson’s complex figure [36] and the spatial span forward [37]; executive functions with the digit span backward [37], and the Modified Card Sorting Test [38]; theory of mind with the Story-based Empathy Task (SET) [39]; language with the token test [40], and phonemic fluency tests [41]; visuospatial abilities with the copy of the Benson’s complex figure [36], and the copy of drawings without landmarks [34], and the presence of behavioral disturbances with the neuropsychiatric inventory (NPI) [42], and the frontal behavioral battery (FBI) [43] administered to patients’ caregivers. In addition, MND and PPA patients performed further neuropsychological tests as previously described [44, 45] and reported in Supplementary Tables 1, 2, respectively.

Healthy controls underwent the same assessment of patients except for FAB and SET. Moreover, in healthy controls the Beck Depression Inventory (BDI) [46] was used to exclude subjects with mood alterations.

### Genetic analysis

Blood samples were collected from all patients and genomic DNA was obtained and processed in each of the recruiting centers. The presence of GGGGCC hexanucleotide expansion in the first intron of the *C9orf72* gene was assessed using fluorescent amplicon-length analysis and a repeat-primed polymerase chain reaction (PCR) assay. A cut-off of ≥30 repeats combined with a typical saw-tooth pattern was considered pathological. In addition, *GRN*, *MAPT*, *TARDBP*, *SOD1*, *FUS*, *TBK1*, *TREM2*, *OPTN* and *VCP* genes were analyzed by Next Generation Sequencing (NGS) and their mutations were confirmed by standard Sanger sequencing. All MND patients were systematically tested for *C9orf72*, *TARDBP*, and *SOD1* mutations, and additional testing of *FUS* and *TBK1* was performed in the presence of positive family history of MND/dementia. Similarly, all other FTLD patients were tested for *C9orf72*, *TARDBP*, *MAPT*, and *GRN* mutations, with additional testing of *FUS, TBK1, TREM2, OPTN* and *VCP* in the presence of positive family history.

### MRI acquisition

Using a 3.0 T scanner (Ingenia CX, Philips), the following brain MRI sequences were obtained from all participants: 3D T1-weighted (TFE) (TR = 7 ms; TE = 3.2 ms; flip angle = 9 [degrees]; 204 contiguous sagittal slices with voxel size = 1 × 1 × 1 mm, matrix size = 256 × 240, FOV = 256 × 240 mm^2^); 3D FLAIR (TR = 4800 ms; TE = 267 ms; TI = 1650 ms; ETL = 167; NEX = 2; 192 contiguous sagittal slices with voxel size = 0.89 × 0.89 × 1 mm, matrix size = 256 × 256, FOV = 256 × 256 mm^2^); 3D T2 (TR = 2500 ms; TE = 330 ms; ETL = 117; NEX = 1; 192 contiguous sagittal slices with voxel size = 0.89 × 0.89 × 1 mm, matrix size = 256 × 258, FOV = 256 × 256 mm^2^); and T2* weighted (GE-EPI) sequence for rs-fMRI (TR = 1567 ms; TE = 35 ms; flip angle = 70; MB = 2; SENSE = 2; FOV = 240 × 240; pixel size = 2.5 × 2.5 mm; slice thickness =3 mm; 320 sets of 48 contiguous axial slices; acquisition time = 8’ and 32”). Before starting the rs-fMRI scanning, the technician talked with the participants through their earphones instructing them to remain motionless, to keep their eyes closed, not to fall asleep, and not to think about anything in particular.

### MRI analysis

MRI analysis was performed at the Neuroimaging Research Unit, IRCCS Scientific Institute San Raffaele, Milan, Italy. The presence of white matter hyperintensities was evaluated on 3D FLAIR and 3D T2-weighted images.

### Voxel-based morphometry

Voxel-based morphometry (VBM) was performed using SPM12 (http://www.fil.ion.ucl.ac.uk/spm/) and Diffeomorphic Anatomical Registration Exponentiated Lie Algebra (DARTEL) registration method [47], to investigate GM volume alterations at a whole-brain level. Details of the VBM pipeline have been described previously [48].

### Resting-state fMRI preprocessing

Rs-fMRI data processing of patients and matched healthy controls, and of young controls was carried out using the FMRIB software library (FSLv5.0) as described previously [49]. The first four volumes of the rs-fMRI data were removed to reach complete magnet signal stabilization. The following FSL-standard preprocessing pipeline was applied: (1) motion correction using MCFLIRT; (2) high-pass temporal filtering (lower frequency: 0.01 Hz); (3) spatial smoothing (Gaussian Kernel of FWHM 6 mm); (4) single-session independent component analysis-based automatic removal of motion artifacts (ICA_AROMA) [50] in order to identify those independent components (ICs) representing motion-related artifacts.

Rs-fMRI data set (‘clean’ from motion-related ICs) were co-registered to the participant’s 3D T1-weighted TFE image using affine boundary-based registration as implemented in FLIRT [51] and subsequently transformed to the Montreal Neurological Institute (MNI) 152 standard space with 4 mm isotropic resolution using non-linear registration through FNIRT [52]. Pre-processed rs-fMRI data for each subject from the young control group were temporally concatenated across participants to create a single 4D data set. This rs-fMRI data set was then decomposed into ICs with a free estimate of the number of components using MELODIC (Multivariate Exploratory Linear Optimized Decomposition into Independent Components) [53]. The resulting young group-IC maps were spatially correlated with a referent atlas of functional ROIs (http://findlab.stanford.edu/functional_ROIs.html), in order to support the visual classification of the most representative functional networks of the brain at rest (i.e., anterior and posterior salience, anterior and posterior default mode [DMN], auditory, sensorimotor, primary and associative visual, basal ganglia, precuneus, visuo-spatial, left and right executive control networks) (Supplementary Fig. 2) [54]. In order to identify the subject-specific temporal dynamics and spatial maps associated with each group IC, a dual regression analysis was applied for all FTLD patients and matched healthy controls [55]. Finally, spatial maps of all participants were collected into single 4D files for each original IC (network) and were ready for the statistical analyses.

### Statistical analysis

Sociodemographic and clinical data were compared between groups using ANOVA models, followed by post-hoc pairwise comparisons, Bonferroni-corrected for multiple comparisons. Neuropsychological data were compared between groups using ANCOVA models, accounting for age, sex and education, and followed by post-hoc pairwise comparisons, Bonferroni-corrected for multiple comparisons. FTLD patients were considered both as a whole group and according to clinical variant and genetic status (i.e., bvFTD, PPA, PSPs, MND, g-bvFTD, g-MND). For all analyses, the threshold of significance was set at *p* < 0.05. The SPSS Statistics 22.0 software was used.

VBM comparisons between all FTLD patients (as well as each FTLD group) and healthy controls were tested in SPM12 using ANCOVA models adjusted for total intracranial volume, age, sex and education. Results were assessed at *p* < 0.05, Family-wise error (FWE)-corrected for multiple comparisons.

The relationships between rs-FC and CATS subtest scores were tested using General Linear Models (GLMs) in FSL (FSLv5.0), including 4D maps for each original IC (network) of patients or healthy controls, separately, as dependent variable, CATS scores as covariates of interest, and age, sex, education and 4D GM coregistered images as nuisance variables. Nonparametric permutation tests (5000 permutations) were used and analyses were restricted within the spatial rs-networks of interest using binary masks obtained by thresholding the corresponding Z map images (Z > 2.3). FWE correction for multiple comparisons was performed, implementing the threshold-free cluster enhancement using a significance threshold of *p* < 0.05.

Mean rs-FC values of spatial clusters that were significantly associated with CATS scores in all FTLD patients at the voxel-wise analysis were obtained by masking 4D maps for each original IC (network) of patients with significant, and IC-correspondent, spatial clusters through FSL (FSLv5.0) tools. Furthermore, a post-hoc analysis (cluster-based) was performed to explore any significant difference between the FTLD groups. Further analyses were performed based on the post-hoc results. Specifically, the effect of genetic cases on the relationships between CATS and rs-FC within visuo-associative and basal ganglia networks was tested excluding them from analysis, as well as the relationship between basal ganglia rs-FC and patient naming scores was explored for FTLD men and women separately. Analyses were adjusted for age, sex and education and assessed at *p* < 0.05, corrected for multiple comparisons, using R Statistical Software (Version 4.0.3; R Foundation for Statistical Computing, Vienna, Austria).

## Results

### Sociodemographic, clinical and neuropsychological features

Sociodemographic and clinical characteristics of healthy controls and FTLD patients as a whole group and stratified according to the clinical diagnosis and genetic status are reported in Table 1. FTLD patients and controls were comparable in terms of age at MRI and education, however they differed in sex, with men being more frequent in FTLD. Compared to controls, the entire FTLD group presented deficits in all investigated cognitive domains, including emotional processing assessed with CATS total and subtests.

The FTLD groups were similar in terms of age, sex and education, and disease duration. Compared to g-bvFTD, PPA and PSPs patients had lower CDR-FTD scores. Concerning cognition and behavior, all patient groups performed similarly at the FAB, spatial memory, verbal comprehension (Token test), theory of mind (SET), and visuospatial abilities. MND and g-MND patients performed better than the other groups in fluency; compared to bvFTD and g-bvFTD cases, they also showed higher performance in global cognition (MMSE) and in emotional processing (CATS), and had less behavioral disturbances according to caregivers’ reports (FBI and NPI). Furthermore, MND patients performed better than the other non-MND patient groups in verbal memory, and better than g-bvFTD in verbal working memory (digit span backward). Finally, compared to g-bvFTD, PSPs patients showed higher performance at the global cognition (MMSE), and, compared to bvFTD, PPA patients had less behavioral disturbances. MND and g-MND patients were similar in all sociodemographic, clinical, cognitive and behavioral investigated domains (Supplementary Table 1).

When stratifying the FTLD sample according to sex, the groups had similar sociodemographic, clinical, cognitive and behavioral features, except for the performance at the CATS affect naming that was lower in men than women (Supplementary Table 3).

### Voxel-based morphometry (VBM)

Compared with healthy controls, FTLD patients showed significant GM volume loss of the bilateral middle and superior frontal gyri, postcentral gyrus, cerebellar Crus II, left insula, middle and posterior cingulate cortices, superior temporal, lingual, fusiform gyri and cerebellar Crus I, and right middle temporal gyrus and supplementary motor area (Supplementary Fig. 3). Compared with healthy controls, each FTLD group showed the expected pattern of brain atrophy (Supplementary Fig. 4) [56–58]. In sporadic and genetic cases of MND, we did not observe GM differences compared to controls [58].

### Resting-state functional connectivity

**Fig. 1:**
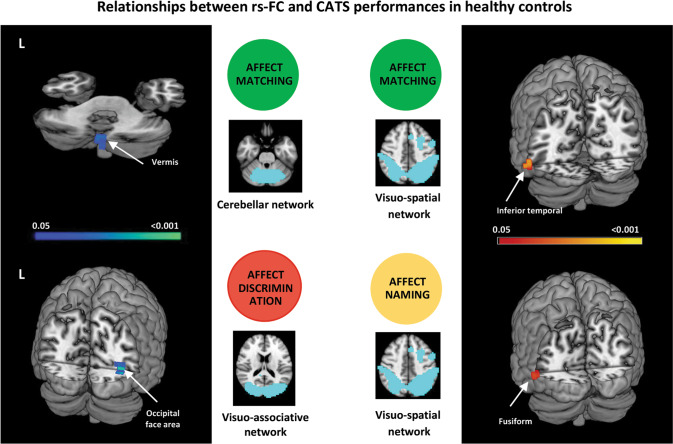
Independent component analysis. Relationship between resting state functional connectivity and CATS scores in healthy controls. Positive and negative relationships are shown in cold and warm colors, respectively. Results are overlaid on the Montreal Neurological Institute (MNI) standard brain and displayed at *p* < 0.05 Family-wise error corrected for multiple comparisons. Age at MRI, sex, education and gray matter density were included in the model as nuisance variables. rs-FC resting state functional connectivity, L Left. Color bar represents *p* values.

In healthy controls, increased rs-FC of the left vermis within the cerebellar network was associated with a better performance at the affect matching subtest, and increased rs-FC of the right occipital face area (OFA) within the visuo-associative network was related with a better score at the emotion discrimination subtest. In the same group, decreased rs-FC of the left inferior temporal and fusiform gyri within the visuospatial network was related with higher score at the matching and naming subtests, respectively (Supplementary Table 4, Fig. 1).

**Fig. 2:**
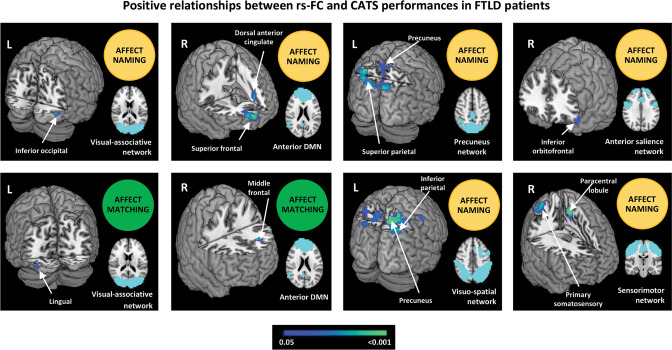
Independent component analysis. Positive relationship between resting state functional connectivity and CATS scores in FTLD patients. Positive relationships are shown in cold colors. Results are overlaid on the Montreal Neurological Institute (MNI) standard brain and displayed at *p* < 0.05 Family-wise error corrected for multiple comparisons. Age at MRI, sex and education were included in the model as nuisance variables. rs-FC resting state functional connectivity, L Left. Color bar represents *p* values.

**Fig. 3:**
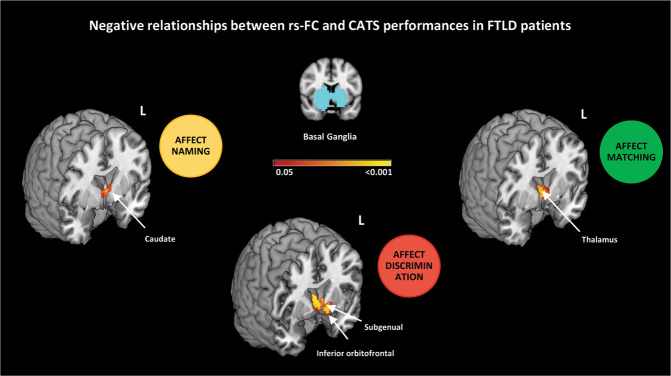
Independent component analysis. Negative relationship between resting state functional connectivity and CATS scores in FTLD patients. Negative relationships are shown in warm colors. Results are overlaid on the Montreal Neurological Institute (MNI) standard brain and displayed at *p* < 0.05 Family-wise error corrected for multiple comparisons. Age at MRI, sex and education were included in the model as nuisance variables. rs-FC resting state functional connectivity, L Left. Color bar represents *p* values.

In FTLD patients, increased rs-FC of the left middle frontal gyrus within the anterior DMN and of the left lingual gyrus within the visuo-associative network were related with a better performance at the affect matching subtest; increased rs-FC of the right superior frontal and left dorsal anterior cingulate within the anterior DMN, of the left inferior orbitofrontal gyrus within the anterior salience network, of the left superior parietal gyrus and right precuneus within the precuneus network, of the left paracentral lobule and right primary somatosensory cortex within the sensorimotor network, of the right precuneus and inferior parietal cortex within the visuo-spatial network, and of the right inferior occipital gyrus within the visuo-associative network were all related with higher scores at the affect naming subtest (Table 2, Fig. 2). On the other hand, in FTLD patients decreased rs-FC of the left thalamus, caudate and subgenual/inferior orbifrontal gyri within the basal ganglia network were related with better performances at the affect matching, naming and discrimination subtests, respectively (Table 2, Fig. 3).

**Table 2:** Significant relationships between CATS subtest performances and resting state functional connectivity within the networks of interest in FTLD patients.

RSN	CATS subtests	Side	Brain regions (BA areas)	N of voxels	MNI coordinate	Post-hoc analysis (cluster-based)				
						Main effect (*p*-values)				Group comparisons (*p*-values)
						Group	Age	Sex	Edu	
Anterior DMN	Matching	L	Middle frontal (BA46)	15	x -22; y 34; z 24	0.876	0.370	0.824	0.704	–
Anterior DMN	Naming	R	Superior frontal (BA10)	141	x 22; y 70; z 12	0.964	0.418	0.742	0.638	–
		L	Dorsal anterior cingulate		x -18; y 46; z 12					
Anterior Salience	Naming	L	Inferior orbitofrontal (BA47)	4	x -54; y 38; z -12	0.521	0.386	0.207	0.806	–
Basal Ganglia^§^	Discrimination	L	Subgenual (BA25)	88	x -2; y 14; z 0	0.068	0.616	0.897	0.305	**MND** ***vs*** **g-bvFTD 0.031**
		L	Inferior orbitofrontal (BA47)		x -26; y 26; z -12					
Basal Ganglia^§^	Matching	L	Thalamus	35	x -2; y -2; z 0	**0.041**	0.680	0.483	0.791	**MND** ***vs*** **g-bvFTD 0.059**
Basal Ganglia^§^	Naming	L	Caudate	10	x -2; y 2; z 4	0.119	0.839	**0.014**	0.456	–
Precuneus	Naming	L	Superior parietal (BA7)	131	x -34; y -66; z 52	0.060	0.553	0.630	0.460	–
		R	Precuneus (BA7)		x 6; y -62; z 64					
Sensorimotor	Naming	L	Paracentral lobule (Primary motor, BA4)	72	x -6; y -26; z 60	0.176	0.382	0.979	0.915	–
		R	Primary somatosensory (BA2)		x 42; y -46; z 68					
VIS-ASS	Matching	L	Lingual (BA18)	5	x -30; y -86; z -12	0.413	0.735	0.102	0.682	–
VIS-ASS	Naming	R	Inferior occipital (BA19)	6	x 38; y -74; z -4	**0.019**	0.294	0.810	0.634	**g-MND** ***vs*** **g-bvFTD 0.025**
Visuo-spatial	Naming	R	Precuneus (BA7)	323	x 6; y -66; z 52	**0.043**	0.754	0.906	0.142	–
		R	Inferior Parietal (BA40)		x 34; y -46; z 48					

Coordinates (x, y, z) are in Montreal Neurological Institute (MNI) space. Results are shown at *p* < 0.05 FWE corrected for multiple comparisons, accounting for age, education, sex and gray matter density. Post-hoc analysis reports the main effect of each variable of interest and comparisons among groups. All findings reported positive relationship between CATS performances and resting state functional connectivity except for those network marked with § showing negative correlations.

*BA* Brodmann area, *bvFTD* behavioral variant of frontotemporal dementia, *CATS* Comprehensive Affect Testing System, *DMN* default mode network, *ECN* executive control network, *MND* Motor Neuron Disorders, *g* genetic, *L* left, *PPA* Primary Progressive Aphasia, *PSP* Progressive Supranuclear Palsy, *R* Right, *RSN* resting state network, *VIS-ASS* visuo-associative (network).

Statistically significant *p*-values are in bold.

**Fig. 4:**
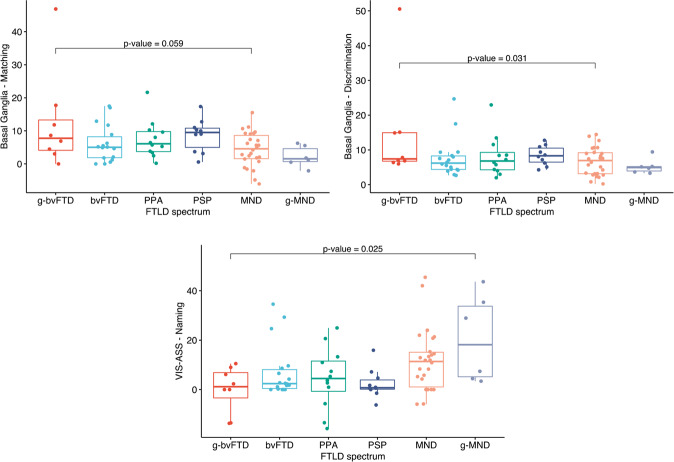
Post-hoc functional connectivity analysis (cluster-based). Mean rs-FC values within the basal ganglia-matching, basal ganglia-discrimination clusters and visuo-associative-naming clusters in FTLD groups. rs-FC resting state functional connectivity, VIS-ASS visuo-associative network, bvFTD behavioral variant of frontotemporal dementia, g-bvFTD behavioral variant of frontotemporal dementia with known genetic mutations, g-MND motor neuron disorders with known genetic mutations, MND Motor Neuron Disorders, PPA primary progressive aphasia, PSP progressive supranuclear palsy.

The post-hoc analysis showed that the rs-FC of the basal ganglia-discrimination cluster was higher in g-bvFTD compared with sporadic MND cases and a trend toward statistical significance was found between these groups also when considering the rs-FC of the basal ganglia-matching cluster (Table 2, Fig. 4). Furthermore, the rs-FC of the visuo-associative-naming cluster was lower in g-bvFTD compared with g-MND (Table 2, Fig. 4). Within the visuo-spatial and the basal ganglia networks, significant relationships between rs-FC and CATS subscores were observed also when all genetic cases were excluded (Supplementary Fig. 5). The post-hoc analysis did not reveal other differences between FTLD groups. Finally, the post-hoc analysis shows a main effect of sex within the basal ganglia-naming cluster (left caudate) where the rs-FC was higher in men compared with women (Table 2; see also Supplementary Table 3 for sociodemographic, clinical and cognitive comparisons between FTLD women and men). Considering FTLD men and women separately, a trend toward a significant relationship between the basal ganglia-naming cluster and naming scores was found in men only (Supplementary Fig. 6).

## Discussion

In the present study we described the pattern of brain resting state functional connectivity related to emotion processing in age-matched healthy controls and a large cohort of FTLD patients. The CATS battery [30] was used as the neuropsychological paradigm for the emotional assessment. We first observed that FTLD patients performed worse than controls in all CATS subtests, confirming their well-known difficulties in emotional recognition [3]. Compared to controls, in FTLD patients rs-FC associated with CATS involved more brain regions and networks, likely reflecting both compensatory attempts and loss of neural specificity. Finally, post-hoc analyses showed that the mean rs-FC values within the majority of the areas correlating with CATS were similar across all FTLD groups, suggesting a common physiopathological mechanism underlying emotion processing deficits, above and beyond patients’ clinical presentation, their genetic profile and the pattern of GM damage.

In healthy controls, increased rs-FC of right vermis within the cerebellar network and of right OFA within the visuo-associative network were related with better emotion matching and discrimination, respectively. Increasing evidence highlights the involvement of cerebellum in cognitive and behavioral processes, including emotions [59]. Importantly, the cerebellum has been shown to be functionally connected with the autonomic nervous system [60]. In patients with lesions or alterations confined to the cerebellum, the dysregulation of affects has been also frequently described [61]. In particular, the vermis would modulate emotion reactions and control emotion expression, and its activation has been shown in functional neuroimaging studies investigating panic, sadness and grief [59]. The recruitment of the right OFA in association with emotion discrimination reflects its role in both face recognition and emotion differentiation [62, 63]. Although previous literature suggested that the OFA has a crucial role in face recognition only [64], more recent evidence has shown that different emotional expressions engage distinct activity patterns in OFA and other face-related areas of the ventral pathway (i.e., the fusiform face area and the superior temporal sulcus) [62, 63]. Indeed, targeting right OFA with repetitive transcranial magnetic stimulation in healthy subjects reduced participants’ accuracy during a facial emotion discrimination task [62]. These findings are consistent with former fMRI evidence suggesting that emotion selective neurons are distributed throughout the ventral temporal lobes [65].

In healthy subjects, we also observed a decreased rs-FC of both left inferior temporal and fusiform gyri within the visuo-spatial network, which was associated with better performances at the affect matching and naming CATS subtests, respectively. In interpreting our findings, the known distinct topographical distribution of the visuo-spatial and the visuo-associative networks should be considered. From previous literature [54] -and confirmed by our rs-FC network representation in Supplementary Fig. 2-, it is evident that the visuo-spatial network involves dorsal regions, which usually subtend high-level visual and spatial processing of the stimuli; on the other hand, the visuo-associative network promotes a more ventral connectivity, including brain regions typically associated with emotion and significance attribution. Thus, we can speculate that in healthy controls the connectivity of brain regions within the visuo-spatial network is reduced in favor of brain circuits specifically dedicated to emotion processing.

Each FTLD group, when compared to controls, showed deficits in all CATS investigated constructs, regardless its clinical presentation (e.g., behavioral, linguistic, or motor) and the specific pattern of atrophy (see Supplementary Fig. 4). As we predicted, among the FTLD conditions, bvFTD and MND cases significantly differed in CATS performances, with the former showing the worse scores and the latter the best ones, well reflecting the two extremes of the spectrum [31], also in terms of social cognition alterations. In some FTLD syndromes, such as bvFTD, the magnitude of social impairment is well-established and has been already included among the core diagnostic criteria [6]. Similar to bvFTD, emotion recognition failures have been demonstrated also in other FTLD variants, such as in svPPA with long disease duration and nfvPPA cases facing low salient stimuli [66, 67], in ALS with and without cognitive disturbances [68, 69], in PSPs across all modalities (facial expressions, prosody, and voice recognition) [70, 71]. Emotional recognition alterations occur in both sporadic and genetic FTLD cases [10]. Despite a number of studies demonstrating the similarity between bvFTD cases (as examples of failure in emotional processing) and one or two other FTLD variants, few works [72] have taken into account the entire FTLD spectrum, including the genetic forms. However, it is important to acknowledge that we used an emotion recognition paradigm, the CATS [30], which investigates several aspects of emotion processing (affect discrimination, naming, and matching) in a short amount of time. Despite these advantages, with using this battery, we could not identify which emotions (among fear, anger, surprise, happiness and sadness) were more frequently mistaken by patients, since the CATS has not been implemented with this purpose. Furthermore, based on the sole facial expression judgement, this battery may fail to detect emotion processing in more ecological situations, such as when patients need to assess changes in emotional valence and arousal. For instance, previous studies suggested that patients with svPPA perform significantly better when tracking the valence of others’ emotions rather than when emotion labeling is required [73]. CATS should be used as a screening tool for detecting emotional processing deficits in suspected FTLD cases, but further tests are warranted to hold a better understanding of the differences among the FTLD variants.

In FTLD patients, the relationships between rs-FC and emotional processing, although present, are more numerous and less specific than those of controls and involve a larger number of networks. Our findings are in agreement with a previous work using a dynamic tracking task of emotion perception (where participants track the changing valence of a film character’s emotions), in which performance of FTLD patients was associated with the integrity of a larger pattern of frontal GM structures than controls, potentially reflecting a larger number of component processes or networks required [73]. Specifically, we observed a positive relationship between the affect naming and matching subscores and the rs-FC of orbitofrontal, superior and middle frontal gyri, and anterior cingulate cortex within anterior brain networks, such as anterior salience and anterior DMN. These findings are not unexpected considering that the salience network includes regions critical for socioemotional processes [74] and that the ability to accurately detect another person’s emotions may also depend on a circuit necessary for social working memory that overlaps with DMN regions [75]. Furthermore, previous research on bvFTD, PSPs, svPPA and nfvPPA showed that damage of more anterior regions, in particular the orbitofrontal cortex, is associated with empathy deficits [76–80]. Interestingly, we observed that the strongest of such relationships were located within the left hemisphere. In a study correlating orbitofrontal cortex damage with behavioral outcomes, authors found that the right ventromedial prefrontal cortex is more directly involved in emotional processing than the left [81]. Also, a number of studies examining patients with unilateral or bilateral orbitofrontal or anterior cingulate lesions suggest that the right medial portions are involved in social emotion processing across input modalities [82, 83]. The left dominant positive relationship we observed in the same regions may be interpreted as a compensatory attempt, likely due to structural and functional alterations of these specialized regions mainly at the right side (which is usually affected earlier that the left during the disease course) [84], and the consequent loss of neural specificity.

Recent rs-fMRI data in bvFTD cases support the idea that two main pathways are implicated in emotional processing in these patients [85]. The first, which involves the anterior temporal lobe, subtends emotion detection and is implicated, for instance, in assessing the level of arousal induced by the stimulus [85]. This task is not investigated by CATS and this is likely the reason for the lack of relationship with the rs-FC of the anterior temporal lobe. On the other hand, the second pathway is dedicated to emotion characterization (e.g., affect naming, matching and discrimination, the emotional domains investigated by CATS in our study) [85]. This latter route involves regions of the action observation circuit, such as inferior frontal, parietal and somatosensory regions, which interchange the emotional information with the limbic system and seem to be the most suffering in bvFTD patients [85]. Our results confirm these previous findings in the entire FTLD spectrum.

Neuroimaging suggests that the structural integrity of caudate and thalamus are important for parsing incoming emotional information to discriminate and understand others’ emotional states [72, 86–90]. In our FTLD patients, basal ganglia were involved in all investigated CATS constructs. The role of basal ganglia in emotional processing is a well acknowledged finding in healthy controls [91] and an association between the integrity of basal ganglia and emotion processing has been observed in different FTLD variants [8]. Specifically, we observed that worse CATS performances related to increased rs-FC in patients. Along these lines, deep brain stimulation (DBS) of subthalamic nuclei (STN) in Parkinson’s disease patients was associated with emotion recognition failure [92]. Accordingly, one study observed that impaired recognition of fear in Parkinson’s disease patients following DBS of STN correlated with reduced glucose metabolism of the orbitofrontal cortex after surgery [93]. This latter study suggests that the role of basal ganglia in emotion recognition is potentially mediated by connections with the orbitofrontal cortices. Thus, in our patients, we can speculate that the disruption of the frontostriatal functional and structural connections could be associated with a dysfunctional increased connectivity of basal ganglia.

In FTLD, the lack of association between emotion processing and rs-FC of cerebellar and specific occipital-temporal structures (such as the right OFA) could be indirectly linked to the degeneration of frontal regions, in particular the prefrontal cortices. Supporting this, different subdivisions of the cerebellum, including the vermis, have been shown to specifically target prefrontal cortices [59]. Also, a more recent neurophysiological study confirmed a causal link between the activity of the prefrontal cortex, which was perturbed by repetitive transcranial magnetic stimulation, and the signal recorded from the OFA during fast emotion discrimination [94].

We then observed that within the basal ganglia circuit, the rs-FC of both the left subgenual, inferior orbitofrontal cortex and thalamus were different in MND sporadic cases when compared to g-bvFTD, with the latter showing higher connectivity. A previous study considering symptomatic and asymptomatic cases sharing the same mutations (i.e., *C9Orf72*, *MAPT*, and *GRN*) showed that low performance at a similar affect facial recognition paradigm was associated with reduced GM integrity of basal ganglia, orbitofrontal and insular cortices [95]. In addition, within the visuo-associative network differences were observed among mutated cases of both MND and bvFTD groups, with the former presenting higher rs-FC of the right inferior occipital gyrus. Occipital cortical alterations and increased rs-FC in the occipital cortex have been already reported in MND [96, 97], in particular *C9orf72* carriers showed hypermetabolism associated with these regions [98]. Notwithstanding the different rs-FC patterns in sporadic and genetic cases, the results did not change when genetic cases were excluded from the correlation analysis within basal ganglia and visuo-associative networks, suggesting a role of these networks in emotion processing across the entire FTLD spectrum.

Interestingly, in FTLD cases, we observed an effect of sex on the association between affect naming and left caudate rs-FC within the basal ganglia network, with men showing higher rs-FC and lower affect naming score than women. Although the higher caudate functional connectivity found in men warrants further investigation, their relatively worse performance in affect naming is a well-known finding [99].

One of the major limitations of our study is the relatively small sample size when patients are stratified according to FTLD groups. Second, due to the cross-sectional nature of the study, the evolving trajectory of emotion recognition deficits in these patients as well as their ability to predict patients’ prognosis should be further investigated. Furthermore, we used an assessment-based paradigm in association to rs-fMRI and not a direct investigation of subjects’ emotions during a task-based fMRI.

To conclude, dysfunction in emotion processing is present across the FTLD spectrum. The CATS paradigm offers a screening tool for detecting early emotional processing changes in each FTLD variant. The relationship between emotion processing and brain functional connectivity is different in FTLD cases and healthy controls, with the former presenting numerous associations resulting from both loss of neural specificity and compensatory attempts. These associations, which mainly include frontal networks, basal ganglia and the action observation circuits, are shared by all FTLD cases, regardless their clinical presentation, genetic status and patterns of GM damage, suggesting a common functional vulnerability pattern linked to emotion processing across the entire FTLD spectrum. The relevance of the present work lies on its potential implications on both the clinical setting and the research field. In all FTLD conditions, not only in bvFTD, having information on emotion recognition failure may support the clinical diagnosis and predict the patient prognosis. Furthermore, this study, which includes both behavioral and imaging data, would help to improve the understanding of the neural networks underlying emotion processing in aging and in neurodegenerative disorders. Brain regions with preserved functioning could be targeted in behavioral interventions (at least in the early stages of the disease).

## Supplementary information

Supplementary material
