# Supplementary material for: Resting state functional brain networks associated with emotion processing in frontotemporal lobar degeneration
Source: Mol Psychiatry. 2022 May 20;27(11):4809–21. doi: 10.1038/s41380-022-01612-9 (PMC9734056; doi:10.1038/s41380-022-01612-9)
Supplement: Supplementary file 1 — Supplementary material [file 41380_2022_1612_MOESM1_ESM.docx]

**S-Table 1.** Clinical and neuropsychological features of MND patients.

|  | **MND** | **g-MND** | **p-value** |
| --- | --- | --- | --- |
| **N** | 26 | 6 | - |
| **Onset [limb/bulbar/limb+bulbar]** | 23/3/0 | 6/0/0 | 0.43 |
| **ALSFRS-r [0-48]** | 37.29 ± 7.68  (22.00 – 48.00) | 40.83 ± 5.15  (32.00 – 46.00) | 0.30 |
| **Disease progression rate** | 1.55 ± 5.14  (0.00 – 23.92) | 0.69 ± 0.93  (0.09 – 2.55) | 0.69 |
| **ECAS** |  |  |  |
| ECAS-Language | 23.54 ± 4.21  (11.00 – 28.00) | 22.67 ± 5.41  (12.00 – 27.00) | 1.00 |
| ECAS-Verbal fluency | 17.33 ± 5.55  (0.00 – 22.00) | 16.00 ± 5.51  (8.00 – 22.00) | 1.00 |
| ECAS-Executive functions | 35.00 ± 9.29  (15.00 – 47.00) | 30.50 ± 10.67  (10.00 – 39.00) | 1.00 |
| ECAS-ALS Specific score | 76.71 ± 17.35  (31.00 – 96.00) | 69.83 ± 16.44  (47.00 – 88.00) | 1.00 |
| ECAS-Memory | 16.92 ± 5.01  (4.00 – 24.00) | 12.83 ± 5.91  (1.00 – 17.00) | 1.00 |
| ECAS-Visuospatial | 11.46 ± 1.06  (8.00 – 12.00) | 11.67 ± 0.52  (11.00 – 12.00) | 1.00 |
| ECAS-Non-specific ALS score | 28.17 ± 5.11  (16.00 – 36.00) | 24.50 ± 6.19  (12.00 – 28.00) | 1.00 |
| ECAS-Total score | 104.29 ± 20.25  (59.00 – 129.00) | 94.17 ± 20.96  (59.00 – 115.00) | 1.00 |
| **Social cognition** |  |  |  |
| CET, Total Score | 14.20 ± 4.66  (3.00 – 25.00) | 15.50 ± 3.39  (11.00 – 19.00) | 1.00 |
| **Mood & Behavior** |  |  |  |
| ECAS-Behavior | 0.75 ± 1.12  (0.00 – 4.00) | 0.00 ± 0.00  (0.00 – 0.00) | 0.30 |
| ECAS-Psychosis | 0.05 ± 0.23  (0.00 – 1.00) | 0.00 ± 0.00  (0.00 – 0.00) | 1.00 |
| HDRS | 5.95 ± 4.26  (1.00 – 19.00) | 2.00 ± 1.83  (0.00 – 4.00) | 0.75 |
| ALS-FTD-Q | 10.05 ± 13.95  (0.00 – 52.00) | 6.20 ± 5.40  (0.00 – 14.00) | 1.00 |

Values are numbers or means ± standard deviations (range). The number of patients performing each test is reported in table. P values refer to ANOVA models or Chi-squared test. Differences in neuropsychological scores were assessed using ANCOVA models, accounting for age, sex and education (Bonferroni-corrected for multiple comparisons). Abbreviations: ALSFRS-r= Amyotrophic lateral sclerosis functional rating scale revised; ALS-FTD-Q= Amyotrophic lateral sclerosis-Frontotemporal dementia-Questionnaire; CET= Cognitive Estimation Test; ECAS= Edinburgh Cognitive and Behavioural ALS Screen; HDRS= Hamilton Depression Rating Scale; MND= Motor Neuron Disease; g-MND= Motor Neuron Disease with genetic mutation; N= Number.

**S-Table 2.** Language assessment administered to PPA patients.

|  | **PPA** |
| --- | --- |
| **N** | 12 |
| CAGI, Naming | 28.55 ± 12.78  (19.00 – 46.00) |
| CAGI, Comprehension | 44.40 ± 3.95  (38.00 – 48.00) |
| PPT, Object knowledge | 41.09 ± 7.08  (29.00 – 51.00) |
| AAT, Repetition | 137.91 ± 21.44  (77.00 – 150.00) |
| AAT, Reading | N 11  28.55 ± 3.86  (17.00 – 30.00) |
| AAT, Dictation | 26.82 ± 5.88  (10.00 – 30.00) |
| ISCT, total score | 41.33 ± 6.64  (29.00 – 48.00) |

Values are numbers or means ± standard deviations (range). The number of patients performing each test is reported in table. Abbreviations: AAT= Aachener Aphasie Test; ICST=Italian Syntax Comprehension Test; N= Number; PPT=Piramids and Palm trees test.

**S-Table 3.** Demographic and clinical features of FTLD patients stratified according to sex.

|  | **FTLD men** | **FTLD women** | **p-value** |
| --- | --- | --- | --- |
| **N** | 47 | 33 | - |
| **Age**  **[years]** | 61.62 ± 12.57  (23.81 – 78.62) | 62.38 ± 8.94  (44.71 – 84.08) | 0.77 |
| **Education**  **[years]** | 11.45 ± 4.13  (5.00 – 28,00) | 11.42 ± 4.46  (5.00 – 23.00) | 0.98 |
| **Disease duration**  **[months]** | 52.00 ± 46.59  (0.46 – 210.96) | 34.72 ± 37.24  (6.60 ± 203.43) | 0.10 |
| **CDR-FTD** | 6.71 ± 5.10  (0.00 – 22.00) | 6.82 ± 6.30  (1.00 – 20.00) | 0.95 |
| **Global cognition** |  |  |  |
| MMSE | 25.80 ± 4.18  (10.00 – 30.00) | 25.85 ± 5.43  (6.00 – 30.00) | 1.00 |
| FAB | 11.32 ± 4.10  (2.00 – 17.00) | 12.18 ± 3.65  (0.00 – 17.00) | 1.00 |
| **Verbal memory** |  |  |  |
| Digit span, forward | 5.20 ± 1.47  (2.00 – 8.00) | 4.97 ± 1.18  (3.00 ± 8.00) | 1.00 |
| RAVLT, delayed recall | 5.98 ± 4.00  (0.00 – 15.00) | 6.58 ± 4.03  (0.00 – 13.00) | 0.99 |
| **Spatial memory** |  |  |  |
| Spatial span, forward | 4.27 ± 1.39  (2.00 – 7.00) | 3.53 ± 1.26  (0.00 – 5.00) | 0.18 |
| Benson’s complex figure, recall | 6.25 ± 4.06  (0.00 – 16.00) | 5.89 ± 3.62  (0.00 – 12.00) | 1.00 |
| Benson’s complex figure, recognition | 0.62 ± 0.49  (0.00 – 1.00) | 0.42 ± 0.51  (0.00 – 1.00) | 0.78 |
| **Executive functions** |  |  |  |
| Digit span, backward | 3.90 ± 1.95  (0.00 – 9.00) | 3.28 ± 1.61  (0.00 – 6.00) | 0.90 |
| MCST, perseverations | 12.05 ± 12.60  (0.00 – 47.00) | 6.52 ± 6.88  (0.00 – 25.00) | 0.15 |
| Attentive matrices | 39.32 ± 12.75  (10.00 – 60.00) | 41.18 ± 12.13  (18.00 – 60.00) | 1.00 |
| TMT B-A  (seconds) | 197.67 ± 144.63  (66.00 – 617.85) | 122.74 ± 63.54  (14.00 –218.00) | 0.30 |
| **Language** |  |  |  |
| Token test | 28.37 ± 5.23  15.50 – 35.00 | 26.63 ± 7.77  5.00 – 36.00 | 0.96 |
| Phonemic Fluency | 19.93 ± 13.67  (0.00 – 59.00) | 26.73 ± 15.13  (0.00 – 55.00) | 0.06 |
| Semantic Fluency | 26.26 ± 13.19  (0.00 – 59.00) | 27.34 ± 15.03  (1.00 – 50.00) | 1.00 |
| **Social Cognition** |  |  |  |
| CATS, Total score | 45.23 ± 7.86  (31.00 – 66.00) | 48.70 ± 8.73  (25.00 – 67.00) | 0.32 |
| CATS, Affect discrimination | 10.55 ± 1.69  (6.00 – 12.00) | 10.58 ± 1.52  (6.00 – 12.00) | 1.00 |
| CATS, Affect naming | 3.00 ± 1.59  (0.00 – 6.00) | 4.06 ± 1.39  (0.00 – 6.00) | **0.02** |
| CATS, Affect matching | 6.70 ± 1.65  (4.00 – 11.00) | 7.33 ± 2.29  (2.00 – 12.00) | 1.00 |
| SET, Global score | 12.07 ± 4.00  (3.00 – 18.00) | 12.67 ± 3.97  (3.00 – 18.00) | 1.00 |
| SET, Intention Attribution | 3.95 ± 1.51  (1.00 – 6.00) | 4.50 ± 1.28  (2.00 – 6.00) | 0.40 |
| SET, Emotion Attribution | 4.05 ± 1.58  (1.00 – 6.00) | 4.17 ± 1.70  (0.00 – 6.00) | 1.00 |
| SET, Causal Inference | 4.05 ± 1.41  (1.00 – 6.00) | 4.07 ± 1.74  (1.00 – 6.00) | 1.00 |
| **Visuospatial abilities** |  |  |  |
| Clock drawing test | 6.03 ± 3.41  (0.00 – 10.00) | 5.52 ± 3.63  (0.00 – 10.00) | 1.00 |
| Benson’s complex figure, copy | 13.41 ± 2.75  (7.00 – 16.00) | 11.79 ± 4.17  (0.00 – 16.00) | 0.36 |
| Copy of drawings without landmarks | 8.70 ± 2.79  (0.00 – 12.00) | 8.76 ± 1.92  (5.00 – 12.00) | 1.00 |
| **Mood & Behavior** |  |  |  |
| FBI A | 10.33 ± 7.95  (0.00 – 24.00) | 7.35 ± 8.16  (0.00 – 27.00) | 0.64 |
| FBI B | 5.34 ± 5.83  (0.00 – 21.00) | 5.08 ± 7.15  (0.00 – 24.00) | 1.00 |
| FBI, Total | 14.59 ± 12.30  (0.00 – 37.00) | 12.27 ± 15.01  (0.00 – 51.00) | 1.00 |
| NPI | 17.55 ± 19.56  (0.00 – 76.00) | 15.44 ± 23.85  (0.00 – 102.00) | 1.00 |

Values are numbers or means ± standard deviations (range). Disease duration was defined as months from onset to date of MRI scan. The number of patients performing each test is reported in table. P values refer to ANOVA models or Chi-squared test. Differences in neuropsychological scores were assessed using ANCOVA models, accounting for age and education (Bonferroni-corrected for multiple comparisons). Disease duration was defined as months from onset to date of MRI scan. Abbreviations: CATS= Comprehensive Affect Testing System; CDR-FTD= Clinical dementia rating scale-Frontotemporal Dementia; FAB=Frontal Behavioral assessment; FBI= Frontal Behavioral Inventory; FTLD= frontotemporal lobar degeneration; MCST= Modified card sorting tests; MMSE= Mini-Mental state examination; N= Number; NPI= Neuropsychiatric inventory; RAVLT= Rey auditory verbal learning test; SET= Story-Based Empathy Task; TMT= Trail making test.

**S-Table 4.** Significant relationships between CATS subtest scores and resting state functional connectivity within the networks of interest in healthy controls.

| RSN | CATS subtests | Side | Brain region | N of voxels | MNI coordinate |
| --- | --- | --- | --- | --- | --- |
| Cerebellar | Matching | L | Vermis | 5 | x -2; y -66; z -44 |
| VIS-ASS | Discrimination | R | Occipital face area (BA19) | 11 | x 34; y -70; z 4 |
| Visuo-spatial^§^ | Matching | L | Inferior temporal (BA20) | 5 | x -54; y -38; z -20 |
| Visuo-spatial^§^ | Naming | L | Fusiform (BA37) | 4 | x -50; y -66; z 0 |

Coordinates (x, y, z) are in Montreal Neurological Institute (MNI) space. Results are shown at p<0.05 FWE corrected for multiple comparisons, accounting for age, education, sex and grey matter density. All findings reported positive relationship between CATS performances and resting state functional connectivity except for those networks marked with § showing negative correlations. Abbreviations: BA=Brodmann area; CATS=Comprehensive Affect Testing System; L=left; R=Right; RSN=resting state network; VIS-ASS= visuo-associative (network).


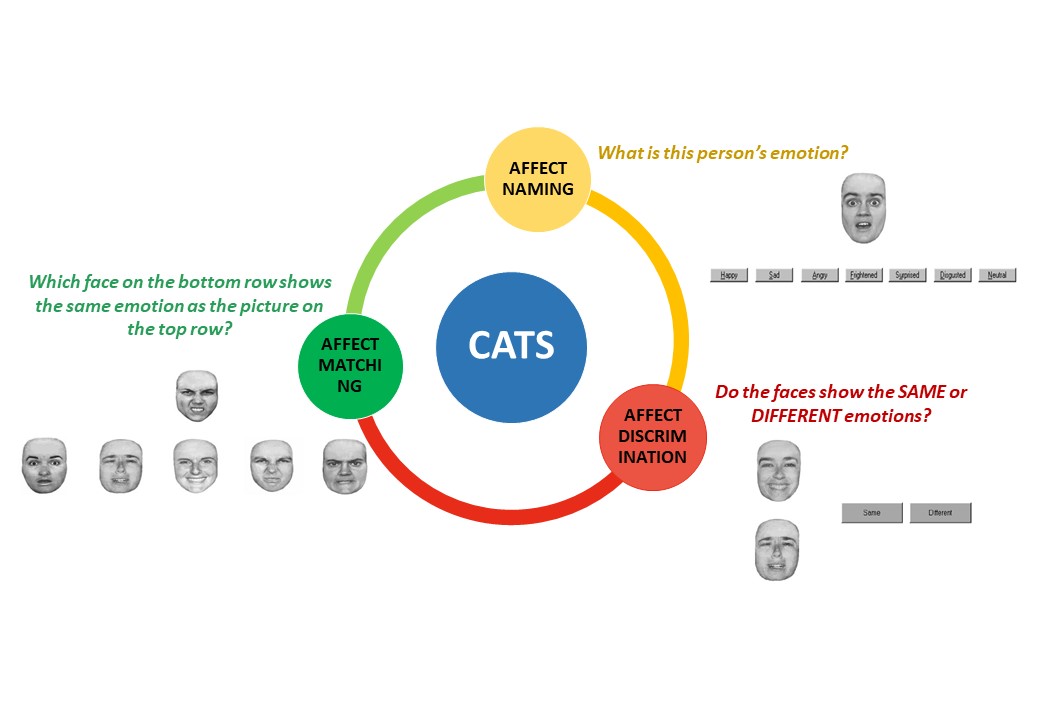


**S-Figure 1.** The subtests of the Comprehensive Affect Testing System (CATS). For illustrative purposes, the stimuli represent an adaptation of the original version.


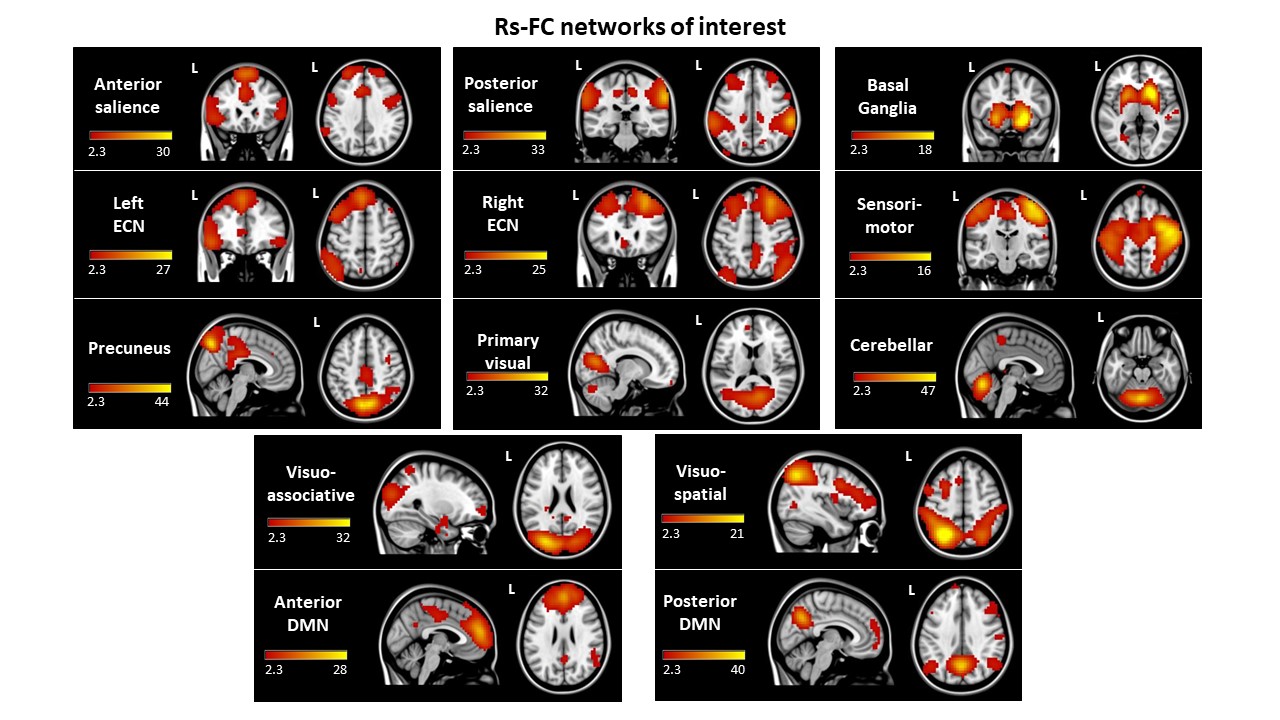


**S-Figure 2.** **Resting state functional connectivity networks of interest.** Results are overlaid on the Montreal Neurological Institute (MNI) standard brain and displayed from Z=2.3 threshold. Abbreviations: rs-FC=resting state functional connectivity; L=Left. Colour bars represent Z values.

**
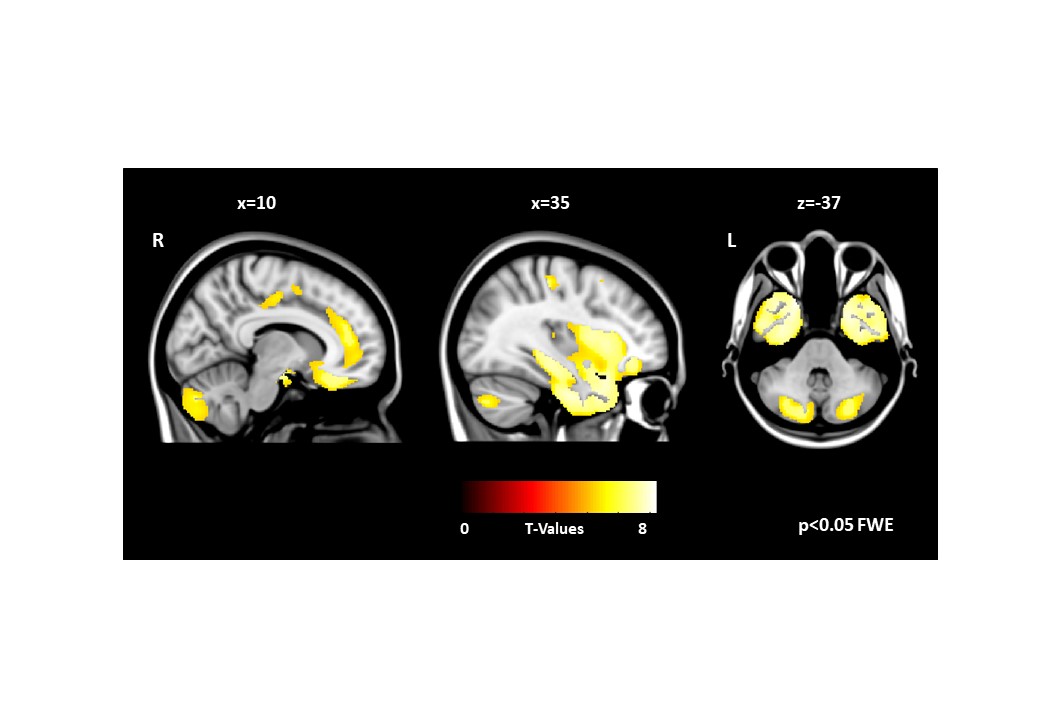
**

**S-Figure 3. Patterns of gray matter atrophy** **in FTLD patients compared to healthy controls.** Results of voxel-based morphometry analysis showing regions of significant gray matter atrophy in all FTLD patients when compared with healthy controls. Significant clusters are overlaid on the sagittal and axial sections of the Montreal Neurological Institute standard brain. Analyses were corrected for age, sex, education and total intracranial volume. Statistical threshold for significance was p<0.05, FWE-corrected for multiple comparisons. L=Left. Coloured bar denotes T-values.


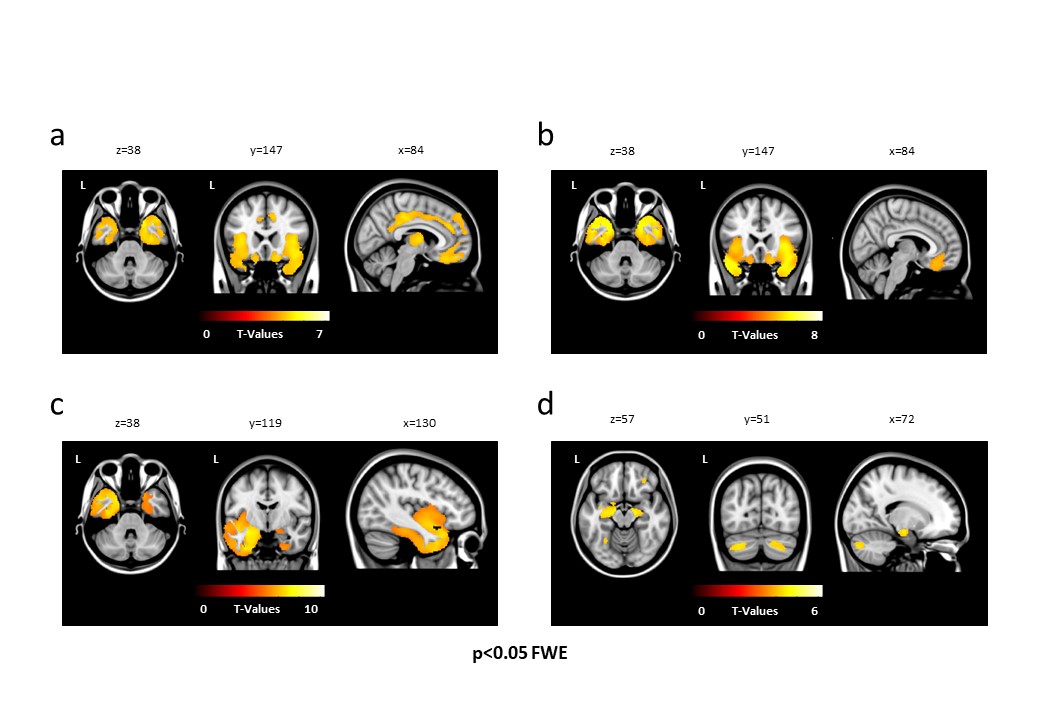


**S-Figure 4. Patterns of gray matter atrophy in each FTLD group compared to healthy controls.** Results of voxel-based morphometry analysis showing regions of significant gray matter atrophy in: a) g-bvFTD *vs* healthy controls; b) bvFTD *vs* healthy controls; c) PPA *vs* healthy controls; and d) PSP *vs* healthy controls. Significant clusters are overlaid on the sagittal and axial sections of the Montreal Neurological Institute standard brain. Analyses were corrected for age, sex, education and total intracranial volume. Statistical threshold for significance was p<0.05, FWE-corrected for multiple comparisons. L=Left. Coloured bar denotes T-values.


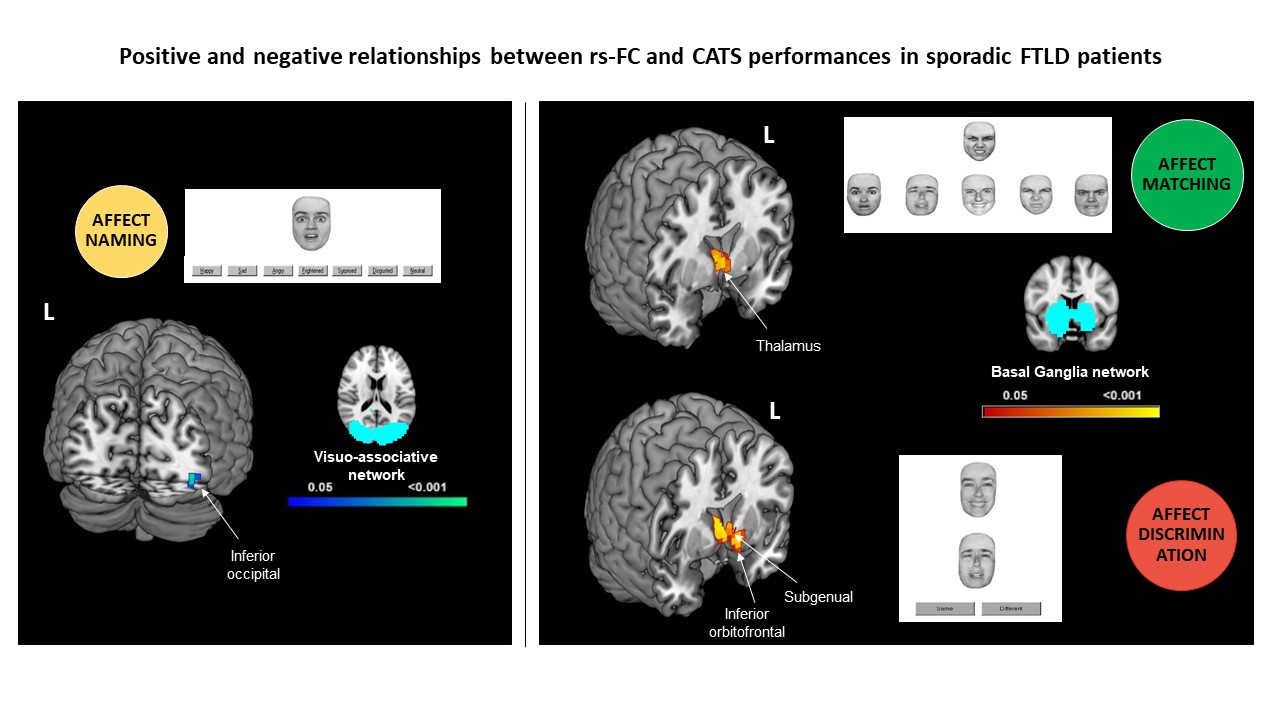


**S-Figure 5.** **Independent component analysis.** R**elationship between resting state functional connectivity and** **CATS scores in sporadic FTLD patients.** Positive and negative relationships are shown in cold and warm colours, respectively**.** Results are overlaid on the Montreal Neurological Institute (MNI) standard brain and displayed at p<0.05 Family-wise error corrected for multiple comparisons. Age at MRI, sex and education were included in the model as nuisance variables. Abbreviations: rs-FC=resting state functional connectivity; L=Left. Colour bar represents p values.

**
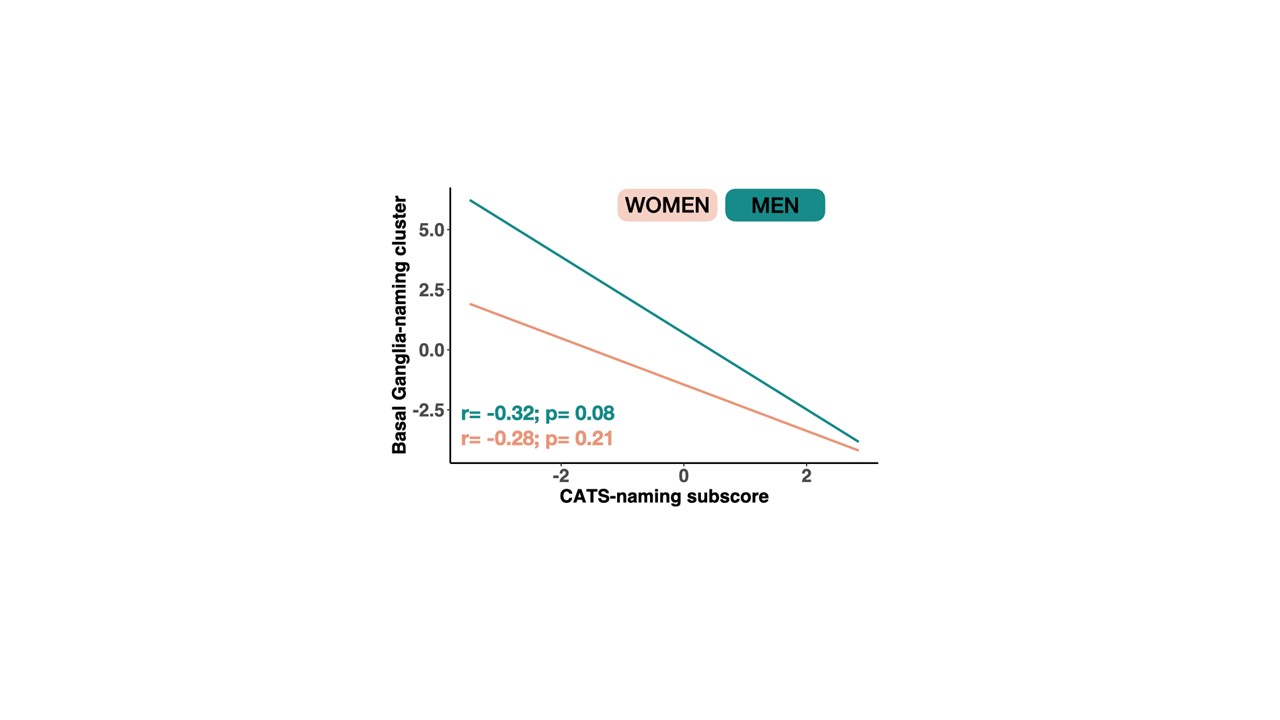
**

**S-Figure 6. Partial residual plot between brain functional connectivity of the basal ganglia-cluster and CATS naming subscore according to sex in FTLD patients.** Results are controlled for age and education. All the variables have been regressed against the covariates and the residuals have been computed. Scatterplot represents the residuals of the two variables of interest after controlling for covariates.
